# Innovative methods for random field establishment and statistical parameter inversion exemplified with 6082-T6 aluminum alloy

**DOI:** 10.1038/s41598-019-54046-9

**Published:** 2019-11-28

**Authors:** Kaikai Zheng, Kangkang Yang, Jun Shi, Jian Yuan, Guangchun Zhou

**Affiliations:** 1 Key Lab of Structures Dynamic Behavior and Control of the Ministry of Education, Harbin Institute of Technology, Harbin, China; 2 School of Transportation Science and Engineering, Harbin Institute of Technology, Harbin, China; 3 Academy of Combat Support, Rocket Force University of Engineering, Xi’an, China; 4 Key Lab of Smart Prevention and Mitigation of Civil Engineering Disasters of the Ministry of Industry and Information Technology, Harbin Institute of Technology, Harbin, China

**Keywords:** Computational methods, Computational science, Civil engineering

## Abstract

This paper aims to eliminate the disharmony between simulation and experiment, and takes the mechanical properties of 6082-T6 Al alloy as an example. In order to obtain the equivalent distribution of material properties after considering the randomicity of materials, a new inversion method combining with stochastic finite element method (SFEM) is proposed. Besides, the discrete random field in SFEM is established by an innovative method to overcome some discretization difficulties in conventional methods. In summary, the generic methods proposed in this study can give a new solution for the correlation of meso-structure and macro-performance in computational materials science.

## Introduction

The numerical simulation of macroscopic material properties plays an important role in material design and application^1^. Undoubtedly, the accuracy of numerical simulation is the focus of attention in computational materials science^2^, and it depends largely on the correctness of input material parameters^3^. However, many materials have the characteristics of heterogeneity, nonlinearity and discontinuity^4^, which makes it difficult to directly measure material properties. Moreover, the stress-strain curve obtained directly from a specimen experiment is not identical to the true constitutive curve, because of the uneven stress distribution caused by the boundary conditions and the large deformation (such as the necking behavior) of the specimen^5^. Therefore, it is necessary to reasonably estimate actual material parameters by inversion methods, which is precisely the parameter estimation problem in inversion theory^6^. For example, some studies combine the finite element method with simple iterative algorithm to calculate the material’s actual stress-strain curve^7^, but on the assumption of homogeneous material.

Thus far, previous studies have failed to consider the randomicity and heterogeneity of material in the inversion procedure. Besides, the inversion result under the homogeneity assumption is applicable only to the particular inversion object and cannot be applied to similar experiments even made of the material of the same batch, due to the material’s natural randomicity. Taking aluminum (Al) alloy as an example, its heterogeneity and randomicity caused by the stochastic meso-structure indeed should not be neglected according to the experimental results of dozens of specimens^8^. Additionally, the meso-structure of materials is commonly simulated by molecular dynamics simulation^9,10^, and it can partially describe the macro-mechanical behavior but usually on the assumption of periodic boundary, which deviates significantly from the real heterogeneous materials with complex spatial distribution^11^. Thus for the simulation of large stochastic structures, such as buildings and airplanes, producing equivalent material properties at macro scale is an effective way, just as the stochastic finite element method (SFEM) does^12^.

An essential part of SFEM is to establish discrete random fields of heterogeneous materials. The conventional means is to approximate random fields with continuous functions, and then descretize them into finite element mesh by the methods of local average, optimal linear estimation, etc^13,14^. However, this is incapable of simulating the failure behavior in high gradient areas, such as strain localization and stress concentration caused by local damage (slip, cracks, voids, etc), as a result of continuity assumption. Moreover, the determination of material’s statistical parameters usually relies on rough estimation and lacks reasonable inference, which should return to the inversion method mentioned above.

Therefore, we propose an inversion method for determining the statistical parameters based on SFEM, in order to obtain the equivalent probability distribution of material properties. Besides, an innovative method directly using finite element mesh to establish random field is also studied, where it constructs stochastic element clusters to imitate model’s meso-structures. In this paper, we take 6082-T6 Al alloy as the application object of the two methods. Plenty of specimens are tested and statistical inference is performed on the experimental results. Besides, the equivalent distribution of 6082-T6 is applied to a box beam experiment to verify the validity and practicability of the methods. By the way, the application of the methods in this paper is not limited to Al alloy.

## Material and Experiments

Al alloy is the most widely used non-ferrous metal material in industry, and it has become the main material for manufacturing transportation tools and light industrial buildings because of good physicochemical properties. And the 6082-T6 (Al-Mg-Si) alloy is the one of the main Al alloys used in building structures due to its advantages of high specific strength, good corrosion resistance, excellent formability and machinability^15^. Accordingly, there are many existing studies on the mechanical properties of 6082-T6^16,17^, but its randomicity lacks research. Thus, plenty of specimen experiments are carried out to study its randomicity.

### Experiments and constitutive model of Al alloy 6082-T6

**Figure 1:**
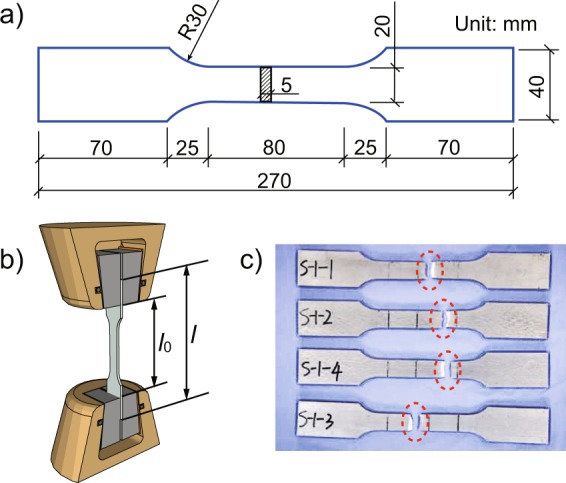
(**a**) Design dimensions diagram, (**b**) experimental device picture and (**c**) final failure picture of Al alloy specimens.

The tensile tests of 6082-T6 alloy are designed according to ISO standard ‘*Metallic materials* — *Tensile testing*’^18^, and Fig. 1(a) gives the dimensions of Al alloy specimens. The tests are carried out on ZWICK electronic universal material testing machine (Fig. 1(b)), and the deformation rate is controlled by the feedback data of extensometer to load slowly. A total of 117 tests for tensile mechanical properties have been carried out, and Fig. 1(c) shows the ultimate failure of some specimens with different fracture locations.

**Figure 2:**
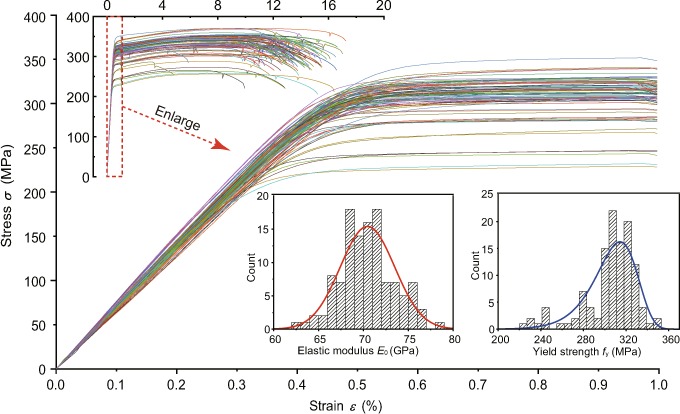
Constitutive curves of test specimens and statistical histograms of material properties.

Then, the stress-strain curves of Al alloy specimens in 117 tests are given in Fig. 2. Besides, the conventional method to calculate the stress *σ* and strain $\varepsilon$ is expressed by the equations

1 $$\sigma =F/{A}_{0};\,\varepsilon =({l^{\prime} }_{0}-{l}_{0})/{l}_{0}$$

where *F* is the applied force and *A*_0_ denotes the area of the middle cross-section. ${l^{\prime} }_{0}$ and *l*_0_ represent the effective length of the test specimens (the distance between the two fixtures) after and before stretching, respectively. It shows that the stress-strain curves of Al alloy are smooth and gradual without a yield platform like low-carbon steel. By the way, the constitutive relation of 6082-T6 obtained by this method is not accurate enough, which will be discussed in the next section. In addition, all the strains used in this paper are of engineering strain.

In order to describe the constitutive relation of Al alloy, many constitutive (stress-strain) models have been put forward. Thereinto, Ramberg-Osgood model is the most widely used one because of its clear physical meaning and nearness to experimental stress-strain curves, and it is shown as follows:

2 $$\varepsilon =\frac{\sigma }{{E}_{0}}+0.002{(\frac{\sigma }{{f}_{0.2}})}^{n}$$

where *E*_0_ is the elastic modulus of Al alloy, and *f*_0.2_ is the stress value corresponding to the plastic strain of 0.2%. The exponent *n* represents the strain hardening degree, which controls the trend of the constitutive curve. And initially, *n* was determined by two parameters: $n=\,\mathrm{ln}\,(2)/\,\mathrm{ln}({f}_{0.2}\,/{f}_{0.1})$ where *f*_0.1_ is the stress with 0.1% plastic deformation. Moreover, Steinhardt^19^ put forward a very simple formula to determine the hardening exponent: $n={f}_{0.2}\,/\,10$ in MPa, ant it has been widely used in many papers. Consequently, the simplified formula is used in this paper to reduce variables, and the constitutive curve of Al alloy can be described by only two parameters *E*_0_ and *f*_0.2_ (also known as yield strength *f*_y_).

### Statistical inference of the experimental results

The statistical histogram of elastic modulus *E*_0_ and yield strength *f*_y_ are illustrated in Fig. 2. The tensile specimen can be regarded as a set of parallel member bars (independent of each other). The elastic modulus *E*_0_ of the whole specimen is the average value of all members’ *E*_0_, so it should be of Gaussian distribution according to the central limit theorem. As for yield strength *f*_y_, a yield member will distribute its force to remaining members, and accelerate the failure of the whole specimen, thus a minimum extreme distribution is suitable for *f*_y_. Moreover, the Anderson–Darling statistical test at the significance level of 0.05 demonstrates that the material parameters *E*_0_ and *f*_y_ follow the Gaussian distribution and Gumbell distribution (extreme value distribution Type-I), respectively.

There are two statistical parameters, location parameter *μ* and scale parameter *δ* of the both types of distribution. And their probability distribution functions are shown as follows:

3 $${P}_{{\rm{E}}}(x)=\frac{1}{\sqrt{2\pi }{\delta }_{{\rm{E}}}}\,\exp (\,-\,\frac{{(x-{\mu }_{{\rm{E}}})}^{2}}{2{\delta }_{{\rm{E}}}^{2}})$$

4 $${P}_{{\rm{f}}}(x)=\frac{1}{{\delta }_{{\rm{f}}}}\,\exp (z-\exp (\,-\,z));\,z=\frac{x-{\mu }_{{\rm{f}}}}{{\delta }_{{\rm{f}}}}$$

where the subscript E and f denote elastic modulus and yield strength, respectively. As for the normal distribution of *E*_0_, the statistical parameters *μ*_E_ = 70.5 GPa and *δ*_E_ = 3.0 GPa can be obtained by the method of moments^20^. In the same way, it can get *μ*_f_ = 305 MPa and *δ*_f_ = 25 MPa for the Gumbell distribution of *f*_y_. By the way, the “*Aluminum Design Manual*” gives a conservative value of 260 MPa of the yield strength of Al 6082-T6^21^.

## Methods

**Figure 3:**
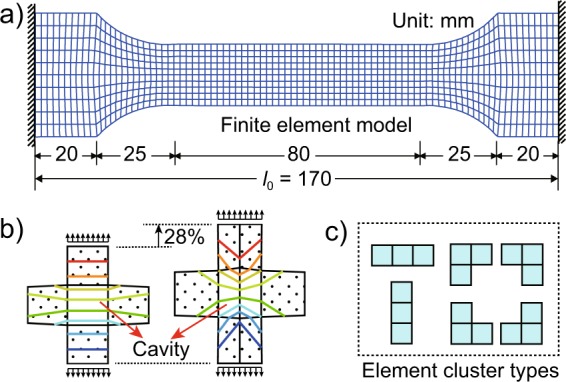
(**a**) Finite element model, (**b**) the displacement contours under different meshing and (**c**) six element cluster types.

However, The stress-strain curves shown in Fig. 2 are the output of experimental specimens rather than the input of material, and they reflect the stress and strain level of the whole specimen instead of every point. Therefore, the stress-stain curve obtained by the conventional method should not be simply deemed to the constitutive relation. For example, a finite element model of the Al alloy specimen (element size 4 mm and total 880 elements) is established by software ANSYS as shown in Fig. 3(a). After extracting the end force and displacement of the specimen from the simulation results, the apparent material properties of the simulated specimen can be output using the same Eq. 1. When assuming the model is of homogeneous material and the parameters *E*_0_ and *f*_y_ take the mode values of the above statistics, the output elastic modulus ${E^{\prime} }_{0}=83.06\,{\rm{GPa}}$ is 17.8% greater than the input *E*_0_, while the output yield strength ${f^{\prime} }_{{\rm{y}}}$ slightly declines by 0.4 MPa. Besides, the influence of finite element discretization on output properties is also studied by changing the element size from 2 mm to 1 mm. It is found that both the ${E^{\prime} }_{0}$ and ${f^{\prime} }_{{\rm{y}}}$ decreased by less than 0.1%, which are much smaller than the discrepancy between input and output. And this discrepancy is mainly caused by the shape and boundary conditions of specimen which makes uneven stress distribution in the specimen. Therefore, the conventional method is not accurate enough and this is also an important reason why the simulated engineering structure, which directly input the apparent stress-stain relationship, is usually more rigid than the actual structure. By the way, all the simulations in this article are of small strain analysis performed in ANSYS, that is, each element uses engineering stress/strain (rather than true stress/strain) which is consistent with the output.

As stated in the introduction, some studies have combined the finite element method with simple iterative algorithm to calculate the actual material properties but on the assumption of homogeneous material. However, according to the failure photograph (Fig. 1(c)) and the statistical material properties of the experimental specimens, the heterogeneous of Al alloy is indeed not negligible. Besides, the distributions in Fig. 2 also cannot actually describe the actual stochastic characteristics of material properties due to the reason proved above. Therefore, an inversion method for obtaining effective material properties needs to be investigated.

### Method for establishing random field

When the material of a finite element model changes from homogeneous to heterogeneous, complex random meso-structures are formed inside the model. And an appropriate random field is necessary to describe the spatial variability of materials in SFEM. One control parameter of random field is the correlation distance^22^, and two points beyond this distance can be considered almost irrelevant. In fact, the value of this distance is often smaller than the size of elements, so all the elements can be considered mutually independent. Accordingly, a direct method is to distribute materials to each element in a purely random way, but chessboard lattice phenomenon is prone to occur in this case. For instance, a five elements model is given in the left side of Fig. 3(b), where a weak element (represents a small cavity) is surrounded by normal elements. And from the vertical displacement contours it can be seen that the cavity element of the original model is unreasonably rigidized compared with the right-side model with a refined mesh.

Therefore, it is necessary to form appropriate element clusters with at least two elements of the same material. Firstly, considering the assumption of isotropy Al alloy, Fig. 3(c) gives six types of element clusters of rotational symmetry. Next, the clusters are randomly distributed as densely as possible in the study area in a mutually exclusive manner, and then each remaining blank element is randomly merged into its adjacent cluster to make the whole study area fully filled. The final step is to assign material properties to element clusters. *E*_0_ or *f*_y_ is sampled randomly from its probability distributions. Consequently, a discrete random field is directly established with finite element mesh by this innovative method which can be called stochastic element cluster method (SEC).

### Inversion algorithm of statistical parameters

Based on the discrete random field established above, the inverse problem of statistical parameters can be described below: Supposing there exists a functional relationship between the input and output material parameter matrices: $g({\boldsymbol{\Lambda }})={\boldsymbol{\Lambda }}^{\prime}$, the inverse problem is to solve ${\boldsymbol{\Lambda }}={{\boldsymbol{\Lambda }}}^{\ast }$ to make $g({{\boldsymbol{\Lambda }}}^{\ast })$ equal to the experimental output matrix ${\boldsymbol{\Lambda }}{^{\prime} }^{\ast }$. The dimension of the matrices is *m* × 2, where *m* denotes the number of material properties and each of them has two statistical parameters. Besides, ${\boldsymbol{\Lambda }}^{\prime}$ is calculated from the following formula:

5 $$\begin{array}{rcl}{\boldsymbol{\Lambda }}^{\prime}  & = & {T}_{l}({[{F}_{i}({{\bf{R}}}_{j})]}_{m\times s});\,l=1,2\\ {{\bf{R}}}_{j} & = & {[{Y}_{k,q};{Y}_{k,q}\sim {P}_{q}(x;{{\boldsymbol{\Lambda }}}_{q,1},{{\boldsymbol{\Lambda }}}_{q,2})]}_{n\times m}\end{array}$$

where ${T}_{l}({\bf{X}}),l=1,2$ represents the estimation of two statistical parameters of each simulated property in the simulation result matrix **X**; ${F}_{i}({{\bf{R}}}_{j})$ is the finite element simulation of the *i*-th output parameter under the random field of the *j*-th random simulation, **R**_*j*_; *s* is the total count of random simulations; *Y*_*k*,*q*_ denotes the random variable of the *q*-th material property on the *k*-th element cluster and it subjects to the probability distribution function ${P}_{q}(x)$, and *n* is the total number of element clusters in random specimens. In addition, ${\boldsymbol{\Lambda }}=[{{\boldsymbol{\lambda }}}_{{\rm{E}}};{{\boldsymbol{\lambda }}}_{{\rm{f}}}]=[{\mu }_{{\rm{E}}},{\delta }_{{\rm{E}}};{\mu }_{{\rm{f}}},{\delta }_{{\rm{f}}}]$ in the specific problem in this paper, and similarly ${\boldsymbol{\Lambda }}^{\prime} =[{\mu ^{\prime} }_{{\rm{E}}},{\delta ^{\prime} }_{{\rm{E}}};{\mu ^{\prime} }_{{\rm{f}}},{\delta ^{\prime} }_{{\rm{f}}}]$.

Unlike conventional inverse problems, the inversion variables in this problem are statistical parameters which need a number of samples to estimate them, and the function relationship between input ${\boldsymbol{\Lambda }}$ and output ${\boldsymbol{\Lambda }}^{\prime}$ is implicit. These results in the uncertain output for a certain input. In view of this, this paper presents an effective iteration algorithm for solving statistical parameters, named as penalized regression secant (PRS), and the algorithm needs to address the following key challenges:

- Function approximation. Regression method is introduced to estimate the implicit function using known iteration points. Besides, the relationship between input and output of each material parameter in this problem is monotonic in theory, so linear regression is enough to guarantee convergence. The regression line of statistical parameter *λ* at the *t*-th iteration can be expressed as $\lambda ^{\prime} ={\beta }^{(t)}\lambda +{\alpha }^{(t)}$.
- Iteration stability. The large fluctuations in estimates of function value could cause abnormal search direction and result in iteration divergence, especially in the initial iteration steps. Therefore, there needs a punishment on the slope angle of regression line when $t=2,3,\ldots ,{t}_{0}$, and the penalty decreases with iteration count rising. Therefore, the coefficients of the regression line becomes::
  6 $$\begin{array}{rcl}|\beta {^{\prime} }^{(t)}| & = & \arctan (\frac{(t-2)\,\arctan (|{\beta }^{(t)}|)+\arctan (1)}{t-1})\\ \alpha {^{\prime} }^{(t)} & = & h(\lambda ^{\prime} )-\beta ^{\prime} h(\lambda ),\,h(x)=\frac{1}{t}\,\mathop{\sum }\limits_{i=0}^{t-1}\,{x}^{(i)}\end{array}$$
- Iteration method. The secant method is a classical and efficient iteration algorithm, so we develop it to approximate the ideal solution $({{\boldsymbol{\Lambda }}}^{\ast },{\boldsymbol{\Lambda }}{^{\prime} }^{\ast })$. Unlike the conventional method, the penalized regression line is applied as the secant and every regression uses all the previous iteration points.
- Simulation times. After repeated trials, it shows that 20 random simulations per iteration are enough to guarantee convergence and convergence faster.
- Solution order. Since the elastic modulus unilaterally affects the yield strength, the yield strength can be calculated subsequent to the solve of ${E}_{0}$ finished.
- Convergence criteria. Since the dependent variable is uncertain, use independent variable to define convergence criterion. For example, when the input parameter vector ${{\boldsymbol{\lambda }}}^{(t)}$ of *E*_0_ or *f*_y_ satisfies $\parallel (2{{\boldsymbol{\lambda }}}^{(t)}-{{\boldsymbol{\lambda }}}^{(t-1)}-{{\boldsymbol{\lambda }}}^{(t-2)})$ $\oslash 2{{\boldsymbol{\lambda }}}^{(t)}{\parallel }_{1}\leqslant \omega$ where $\oslash$ denotes Hadamard division and $\parallel {\boldsymbol{x}}{\parallel }_{1}$ is the Taxicab norm, it can be considered convergent at the *t*-th iteration.

In addition, the pseudo-code of the PRS algorithm for solving statistical parameters is given in the Appendix.

## Results

**Figure 4:**
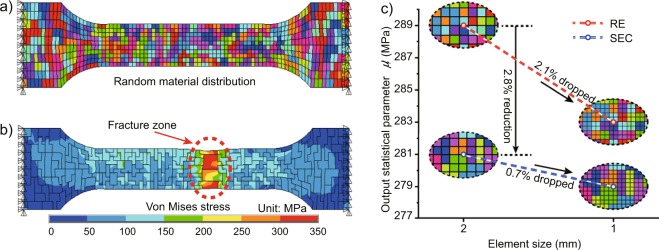
(**a**) Random distribution of element clusters distribution, (**b**) contours of Von Mises stress and (**c**) the discretization errors of the two methods.

As a result, Fig. 4(a) shows a set of random material distribution obtained from SEC, and it can be seen that there are no isolated elements except at the boundaries of the study area. After simulating, its contour diagram of Von Misses stress is shown in Fig. 4(b). Moreover, the failure mode is similar to the experiment results shown in Fig. 1(c), which illustrates the effectiveness of the discrete random fields established by SEC. Taking the experimental statistics result (shown in Fig. 2) as the input material distribution, the discretization errors of random element method (RE) and SEC are shown in Fig. 4(c) by changing element size from 2 mm to 1 mm. Each value in the figure is the location parameter of 50 random simulation results, ${\mu ^{\prime} }_{{\rm{f}}}$. It can be seen that the undesirable rigidification of chessboard lattice is alleviated in SEC. And the lower drop of SEC with mesh refining indicates that the assembly of element clusters can significantly reduce the discrete error.

In summary, the SEC method can obtain excellent simulation results with fewer elements, and it can manually set the shape of element cluster types to imitate specific meso-structures, such as the layered material. Considering the computational efficiency, the number of elements (average 3.5) within each cluster in this paper is sufficient for accurate simulation. Moreover, there exists a considerable gap between the input and output distribution of material properties, for example, the location parameter of ${f^{\prime} }_{{\rm{y}}}$ is 281 MPa (given in Fig. 4(c)) while that of input *f*_y_ is 305 MPa. Therefore, the PRS inversion method is performed to obtain the equivalent input parameters of material distribution.

**Figure 5:**
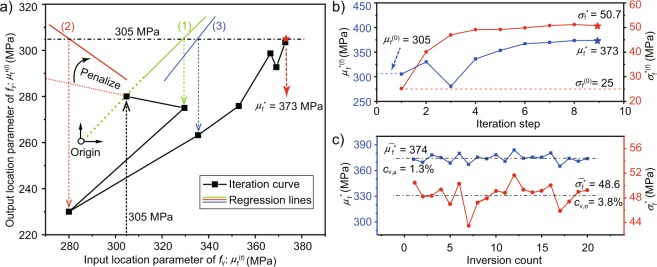
(**a**) Inversion process of the PRS algorithm, (**b**) convergence curves and (**c**) 20 repeated inversion results of the statistical parameters of *f*_y_.

The inversion process of *f*_y_ is shown in Fig. 5(a), and the regression lines of the first three steps are also shown in the figure. And the line of iteration pairs $({\mu }_{{\rm{f}}}^{(t)},{\mu ^{\prime} }_{{\rm{f}}}^{(t)})$ converges to (373, 305) MPa. From the second iteration step, it can be seen that punishing the slope of the regression line can effectively control the serious deviation of the search direction. Besides, the convergence curves of ${\mu }_{{\rm{f}}}^{(t)}$ and ${\delta }_{{\rm{f}}}^{(t)}$ are shown in Fig. 5(b), and they all converge stably and efficiently. Moreover, after 20 repeated inversion, the average of inversion results can be considered as a more accurate solution, Λ* = [61.4 GPa, 12.7 GPa; 374 Mpa, 48.6 Mpa]. Figure 5(c) shows the twenty inversion results of *f*_y_. The maximum coefficient of variation *C*_v_ is less than 4%, which indicates the effectiveness of the algorithm, while that of *E*_0_ is smaller.

In addition, the influence of element size to the inversion results is also studied. As listed in Table 1, with the decrease of element size, the ${\mu }_{{\rm{E}}}^{\ast }$ fluctuates slightly while the other three variables have an increasing trend with varying degrees. The material distributions with smaller elements have higher dispersion, which is precisely the reverse process of homogenization from meso to macro. As a result, the element size largely affects the inversion results, which can be determined by considering computational efficiency and simulation accuracy. By the way, the size of element clusters should be greater than the correlation distance.

**Table 1:** Relationship between statistical parameter inversion results and cell size.

Element size (mm)	Elastic modulus (GPa)		Yield strength (MPa)	
	${\bar{{\boldsymbol{\mu }}}}_{{\bf{E}}}^{\ast }$	${\bar{{\boldsymbol{\delta }}}}_{{\bf{E}}}^{\ast }$	${\bar{{\boldsymbol{\mu }}}}_{{\bf{f}}}^{\ast }$	${\bar{{\boldsymbol{\delta }}}}_{{\bf{f}}}^{\ast }$
5	61.39	12.03	358.5	38.3
2.5	61.43	12.57	374.2	42.9
2	61.36	12.68	381.6	48.6
10/7	61.38	13.11	392.7	55.1
1	61.42	13.19	402.1	62.0

In summary, the SEC method can qualitatively describe the stochastic characteristic of specimens using element cluster to imitate meso-structure. Furthermore, the equivalent material statistical input parameters are obtained by the PRS inversion algorithm, which further quantitatively ensures that random simulations can accurately reflect the macro-mechanical behaviors of the specimens. In addition, the SEC method can not only simulate the stress behavior in high gradient regions, but also facilitate the processing of irregular curved surfaces and non-Gaussian fields which are hard for conventional methods^23^.

## Application

**Figure 6:**
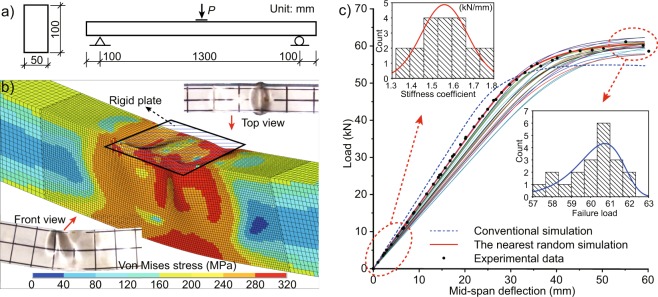
(**a**) Design dimensions of the box beam experiment, (**b**) simulation result compared with experimental photographs and (**c**) statistical analysis of 20 groups random simulation.

Obtained from the above two methods, the equivalent stochastic constitutive relation and material spatial distribution can accurately reflect the statistical regularity of the properties of 6082-T6 material. Moreover, it can be applied to larger structures composed of the same material. For example, Fig. 6(a) shows a box beam of 6082-T6 Al alloy with the cross-section of 50 × 100 × 4 (width, height and thickness in millimeter). The beam model with a length of 1.5 m is loaded at three points and it has 100 mm margin on both sides. Then, a finite element model is built accordingly to analyze the mechanical properties of the experimental beam. Loading device of the experiment is simulated accurately by establishing a rigid plate and reasonable contact as shown in Fig. 6(b). In order to guarantee simulation accuracy and improve computational efficiency, fine grids with 2.5 × 2.5 mm^2^ are divided into the middle part and 5 × 5 mm^2^ in the other parts. The corresponding statistical parameters of these two sizes are listed in Table 1, respectively. In addition, the first and second order buckling modes with the amplitude of 3 mm are added to the model as initial geometric imperfections.

Figure 6(b) shows the Von Mises stress contours and deformation of one random simulation. It can be seen that its local buckling is very similar to the experimental photographs of the top and front views, which shows the effectiveness of the simulation. Besides, 20 groups of random simulations are carried out and their simulated load - displacement curves are shown in Fig. 6(c). And it can be found that a simulated curve (red solid line) which is very close to the experimental data (black dots). Furthermore, statistical analysis of stiffness coefficient and failure load of the structure (corresponding to elastic modulus and yield strength of the constitutive curve) shows that they still maintain normal distribution and extreme value distribution respectively. As a comparison, the conventional simulation is conducted which applies average values of experimental data of specimens as the constitutive parameters. Figure 6(c) gives the simulation result and it can be seen that the elastic part is obviously stiffer than the experiment while the failure part is lower. Therefore, the correct constitutive parameters obtained from the proposed methods can solve the common problem in engineering that the simulated structure more rigid than the real structure.

Moreover, the accurate random simulations can replace repeated experiments, and it can greatly save experimental cost. In addition, the statistical analysis of the simulated results can provide an accurate and direct basis for the reliability design of engineering structures. Furthermore, the input parameters of other physical properties (such as thermal conductivity, electrical conductivity, etc.) can also be inverted by the methods.

## Conclusion

In order to make the random simulation of specimens consistent with the experimental data, the SEC method for constructing discrete random fields and the PRS iteration algorithm for statistical parameter inversion are established. Besides, the two new methods can effectively solve the key problems in SFEM: random field discretization and parameter determination. And they together ensure that the element clusters are equivalent to the random meso-structures of specimens, both qualitatively and quantitatively. And the equivalent stochastic material distribution can be used to accurately simulate larger random structures of the same material. In addition, other physical properties of materials can also apply the methods to connect meso-structure and macro-performance.

## Figures and Tables

**Algorithm 1:**
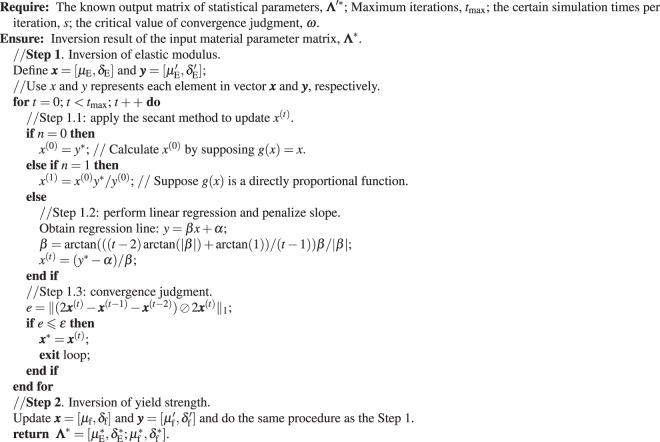
The iterative algorithm, penalized regression secant (PRS), for the inversion of statistical parameters.
